# On the Antimicrobial Potential of *Asparagopsis armata*’s Ethanol Extract: A New Multiple-Industry Bio-Product?

**DOI:** 10.3390/ijms262311358

**Published:** 2025-11-24

**Authors:** Rafael Félix, Pedro Dias, Adriana P. Januário, Carina Félix, Andreu Blanco, Filipa Amaro, Paula Guedes de Pinho, Patrícia Valentão, Marco F. L. Lemos

**Affiliations:** 1 MARE-Marine and Environmental Sciences Centre & ARNET—Aquatic Research Network Associated Laboratory, ESTM, Polytechnic of Leiria, 2520-641 Peniche, Portugal; pedrodavidmartinsdias@gmail.com (P.D.); adriana.p.januario@ipleiria.pt (A.P.J.); carina.r.felix@ipleiria.pt (C.F.); marco.lemos@ipleiria.pt (M.F.L.L.); 2 REQUIMTE/LAQV, Laboratório de Farmacognosia, Faculdade de Farmácia, Universidade do Porto, 4050-313 Porto, Portugal; valentao@ff.up.pt; 3 Centro de Investigación Mariña, Future Oceans Lab, Universidade de Vigo, 32310 Vigo, Spain; andreu.blanco@gmail.com; 4 Associate Laboratory i4HB-Institute for Health and Bioeconomy, University of Porto, 4050-313 Porto, Portugal; filipa_amaro@hotmail.com (F.A.); pguedes@ff.up.pt (P.G.d.P.); 5 UCIBIO—Applied Molecular Biosciences Unit, Laboratory of Toxicology, Faculty of Pharmacy, University of Porto, 4050-313 Porto, Portugal

**Keywords:** bioprospecting, bioactive compounds, marine natural products, antimicrobial resistance, antimycotic, antibacterial

## Abstract

The identification and development of novel antimicrobials is a crucial challenge in the face of increasing antibiotic and antimycotic resistance. As such, there is growing interest in exploring the chemical diversity of natural sources, such as invasive seaweeds such as *Asparagopsis armata*. The valorization of such sources can further contribute to the development of bio-based industries, aligning with societal goals for environmental and economic sustainability. Therefore, a solid-liquid extraction method was performed using ethanol, and the obtained extract was studied for chemical composition elucidation, bioactivity, and toxicity evaluation. Analysis by GC-MS revealed some major chromatographic peaks, including floridoside (2-α-O-D-galactopyranosyl glycerol), glycerol, and oleamide. Also, several other smaller peaks were tentatively attributed to Low Molecular Weight Carbohydrate Derivatives, including isosaccharino-1,4-lactone, which had only been reported once in nature. The extract demonstrated significant antioxidant activity as measured by Ferric Reducing Antioxidant Potential and Oxygen Radical Absorption Capacity, but not by Lipid Peroxidation Inhibitory Potential assays, which is in line with its polar nature. The extract exhibited antimicrobial activity against various microorganisms, with a MIC of 2 mg/mL observed for *Staphylococcus epidermidis*, *Vibrio parahaemolyticus*, and the three yeast strains tested. Moreover, the extract inhibited the growth and phenotypic changes in filamentous fungi, which may result in reduced virulence. Specifically, the extract inhibited sporulation in *Aspergillus fumigatus* and orange pigmentation in *Fusarium graminearum*, possibly by a reduction in the production of aurofusarin, rubrofusarin, and mycotoxins. In vitro cell viability assays in 3T3, RAW264.7, and HaCaT demonstrated the extract was not cytotoxic or presented low cytotoxicity at concentrations up to 0.1 mg/mL, but a strong cytotoxic effect was observed at 1 mg/mL. At non-cytotoxic concentrations, the ethanol extract inhibited up to 48% of NO production in LPS-stimulated RAW264.7. This may indicate that anti-inflammatory activity may add to the antimicrobial activity in human and animal systemic and topical applications of the extract. In this work, new molecules were reported in *A. armata,* and the bioactivities reported were novel for this extract and algal species—especially through the choice of uncommon but very relevant pathogens to study. Our findings are a valuable contribution to addressing challenges in human and animal health, food and feed technology, as well as animal husbandry and agriculture.

## 1. Introduction

Researchers and stakeholders are increasingly interested in marine natural products for a range of applications [1]. One area of research that holds promise for creating a more sustainable bioeconomy is blue biotechnology, which also aims to reduce dependence on petrochemicals. To achieve this goal, novel ingredients derived from marine biomass must be explored, such as extracts or compounds, which have been shown to possess various bioactivities, including antimicrobial, antioxidant, antitumoral, antiviral, anti-fouling, anti-diabetic, and more [1].

To ensure the viability of bioprospecting and blue biotechnology, the biomass used (the source of the extracts and compounds) needs to be sustainable to either harvest from nature or produce. A promising approach to address this issue involves the valorization of invasive species’ biomass, which offers the dual benefits of creating a bio-based industry and promoting localized ecosystem remediation [2]. However, this strategy faces a significant hurdle in the form of legislative blockages that restrict the use of invasive species biomass, despite the scientific community’s consensus that allowing its valorization can be an effective means of controlling biological invasion [3]—provided sufficient specific and strong management actions are employed to avoid bad practices that may actually have the opposite effect of spreading the problem for the benefit of the industry.

Among marine biomass, seaweeds in general, and invasive seaweeds in particular, are, for those reasons, especially interesting biomass sources to explore. *Asparagopsis armata* (Harvey, 1855) is an invasive red seaweed, which is highly abundant in Europe, originating in New Zealand [4]. It has a life cycle with three main stages: the gametophyte, which has the algae’s typical branches and vegetative growth as well as sexual speciation; the carposporophyte, which occurs when the female gametophyte is fecundated; and the tetrasporophyte, a free-living form of the algae, also known as *Falkenbergia rufolanosa* ((Harvey) F. Schmitz, 1897), that is formed in winter and originates new gametophytes [5]. While some potential has been demonstrated for *F. rufolanosa*, most studies on bioactivities have focused on the gametophyte/carposporophyte, as they provide larger specimens and more biomass.

Recently, the chemistry, bioactivities, and market potential of *A. armata* have been reviewed [5]. This body of work suggests that *A. armata* is primarily studied for its antioxidant and antibacterial properties, which it exhibits effectively. However, its antimicrobial potential remains underexplored. For instance, the possibility of obtaining industry-compatible extracts that might be introduced in the market as functional ingredients against food and feed-borne contaminants (with implications in shelf life and human/animal health), veterinary and phytopathogens, and human pathogens should be explored in depth against currently relevant species of bacteria and fungi.

In this study, an industry-compatible extract of *A. armata*’s gametophyte was produced and characterized both chemically and functionally. Specifically, an ethanol extract was obtained using previously optimized conditions [6], and its chemical profile was analyzed. The extract was then tested against several economically relevant species of microbes, including *Bacillus cereus*, *Staphylococcus aureus*, *Staphylococcus epidermidis*, *Escherichia coli*, *Cutibacterium acnes*, *Vibrio parahaemolyticus*, *Streptococcus uberis*, *Aspergillus fumigatus*, *Candida albicans*, *Cryptococcus neoformans*, *Fusarium graminearum*, *Malassezia furfur*, *Tricophyton rubrum*, and *Stemphylium vesicarium*. Cell viability with mammalian skin cell lines (3T3 and HaCaT) and nitric oxide production inhibition in an in vitro mammalian inflammation model (lipopolysaccharide-stimulated RAW 264.7 macrophages) were also assessed to preliminarily evaluate cytotoxicity and anti-inflammatory potential, further potentiating future research and, ultimately, biomass valorization.

## 2. Results

The ethanol extract from AAG was produced in triplicate. The yield of extraction was 1.01% (W/W based on dry weight) ± 0.10 (SEM). The extract was analyzed by GC-MS after silylation, and the Total Ion Chromatogram (TIC) can be found in Supplementary Material (Figure S1). Sixty peaks were checked for tentative identification by comparison of the fragmentation pattern with spectral libraries, as well as of the retention indices, and 17 of those were included in the manuscript (Table 1) once they could be assigned to either a defined chemical structure (6/17) or, at least, to a narrow class of compounds (11/17). These later 11 peaks all shared a mass spectral signature that indicated the presence of at least one monosaccharide derivative moiety, even though a definitive identification cannot be attempted due to the structural and spectral similarity between different simple sugar derivatives. For that reason, they were all classified as Low Molecular Weight Carbohydrate Derivatives (LMWCDs). The mass spectra of floridoside and of the compound at 15.859 min in the chromatogram obtained are presented in Figure S2.

The antioxidant activity of AAG ethanolic extract is present in Table 2. The value of LPIP activity was low (11.5% inhibition), compared to the significant values of antioxidant activity in the most hydrophilic assays, FRAP (1.96 mM eq. Fe^2+^/mg extract) and ORAC (15.75 µmol eq. Trolox/g extract).

**Figure 1:**
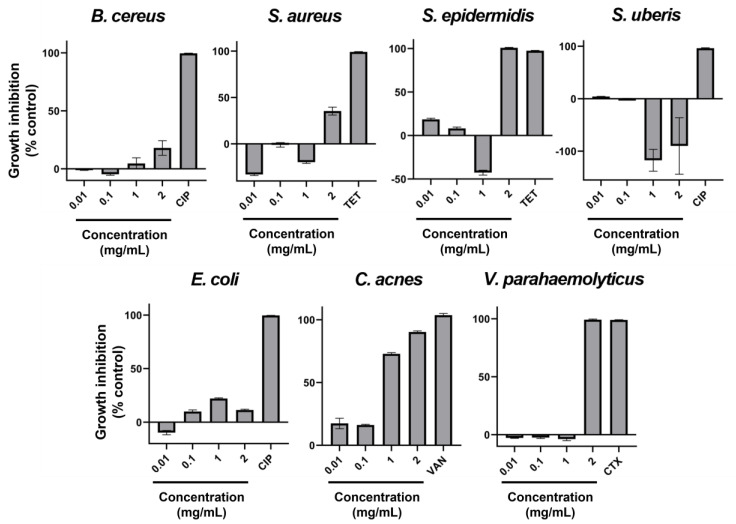
Percentage of growth inhibition with AAG ethanol extract increasing concentrations (0.01, 0.1, 1, and 2 mg/mL), as well as a positive control of inhibition (antibiotic) for *Bacillus cereus*, *Staphylococcus aureus*, *Staphylococcus epidermidis*, *Streptococcus uberis*, *Escherichia coli*, *Cutibacterium acnes*, and *Vibrio parahaemolyticus*. Error bars in each datapoint are the calculated SEM (*n* = 3). CIP—Ciprofloxacin; TET—Tetracycline; VAN—Vancomycin; CTX—Cefotaxime.

Seven very relevant species of bacteria that pose some sort of challenge to different industries have been tested concerning their susceptibility to the ethanolic extract of AAG. The results of the microdilution assays conducted can be found in Figure 1. Overall, despite the occurrence of mixed dose-response curves, it can be observed that *Staphylococcus epidermidis*, *Cutibacterium acnes,* and *Vibrio parahaemolyticus* have the highest susceptibility to AAG ethanol extract. It is also apparent that this extract promotes the growth of *Streptococcus uberis*.

**Figure 2:**
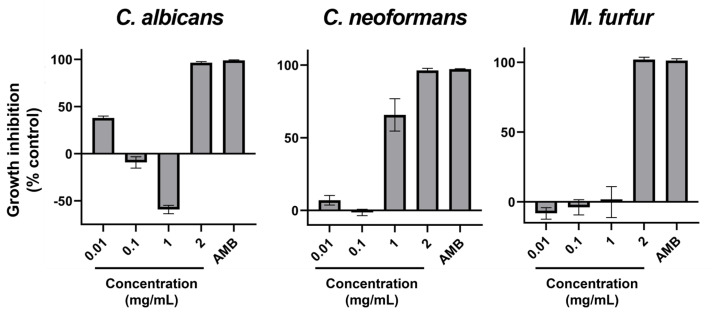
Percentage of growth inhibition with AAG ethanol extract increasing concentrations (0.01, 0.1, 1, and 2 mg/mL), as well as a positive control of inhibition (antimycotic) for *Candida albicans*, *Cryptococcus neoformans*, and *Malassezia furfur*. Error bars in each datapoint are the calculated SEM (*n* = 3). AMB—Amphotericin B.

Similar to bacteria, yeast-like fungi have been assayed for their susceptibility to ethanolic extract of AAG. In this case, three species of yeasts that affect human and animal health to a great extent have been chosen. The results of the adapted microdilution assays are present in Figure 2. Despite some irregularities in dose-response, all yeasts tested were susceptible to AAG ethanol extract, all presenting MIC at the highest concentration tested (2 mg/mL).

Four species of filamentous fungi with high relevance in human and animal health (*Aspergillus fumigatus* and *Tricophyton rubrum*) and the agriculture industry—and consequently the food and feed industries—(*Stemphylium vesicarium* and *Fusarium graminearum*) were tested regarding their susceptibility to AAG ethanol extract by the Poisoned Food Technique, a method that allows both inhibition quantification and phenotype investigation upon exposure to the extract. In Figure S3, all data regarding inhibition (in millimeters) for all concentrations is shown.

As can be seen, only for the higher concentration tested (2 mg/mL), a significant and quasi-linear response in inhibition size (in millimeters) was obtained for the 4 microorganisms tested. Thus, by calculating the linear regression for this concentration (and for the controls) for each microorganism, one can obtain the slope of each line, which then allows the calculation of a measure of how much the growth rate of the fungus (in micrometers per hour, µm/h) was slowed down by the extract (called here the Growth Rate Slow Down Factor, GRSDF). Table 3 provides the GRSDF’s mean and 95% Confidence Interval (95CI), as well as the growth rates obtained for each species in the absence of extract, and finally, an inhibition percentage calculated from these two.

**Figure 3:**
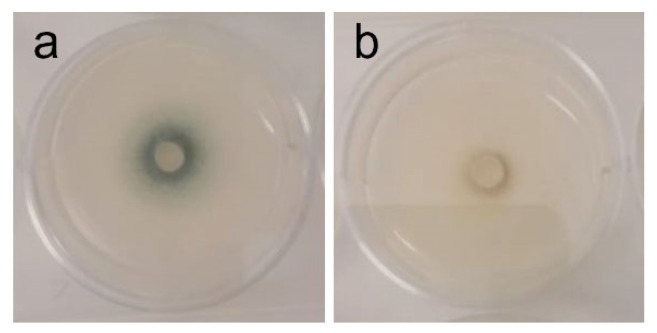
Phenotypic alterations in *Aspergillus fumigatus* in the presence of AAG ethanol extract at full radial growth [(**a**)—control top view; (**b**)—ethanol SLE top view].

**Figure 4:**
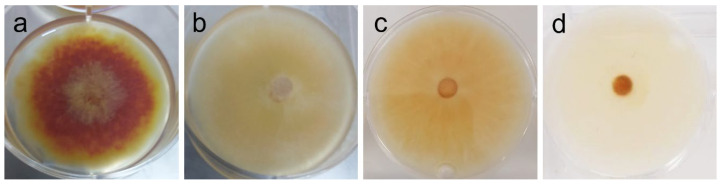
Phenotypic alterations in *Fusarium graminearum* in the presence of AAG ethanol extract at full radial growth [(**a**)—control top view; (**b**)—vehicle (2% *v*/*v* DMSO) control top view; (**c**)—vehicle (2% *v*/*v* DMSO) control bottom view; (**d**)—ethanol SLE bottom view].

**Figure 5:**
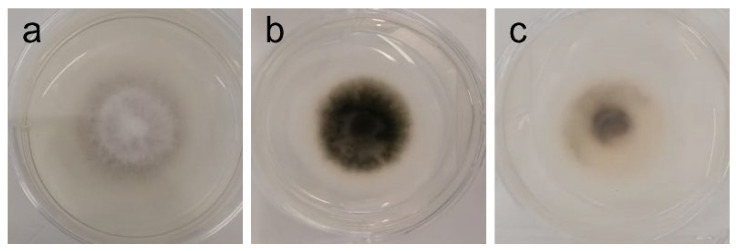
Phenotypic alterations in *Stemphylium vesicarium* in the presence of AAG ethanol extract at full radial growth [(**a**)—control top view; (**b**)—control bottom view; (**c**)—ethanol SLE bottom view].

**Figure 6:**
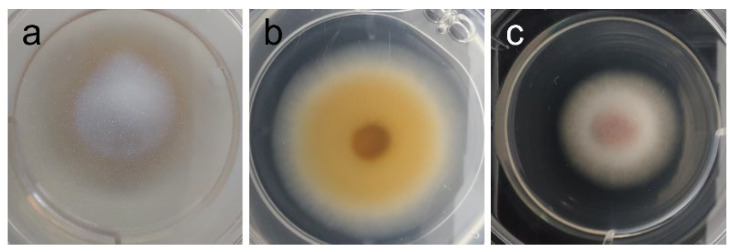
Phenotypic alterations in *Tricophyton rubrum* in the presence of AAG ethanol extract [(**a**)—control top view; (**b**)—control bottom view; (**c**)—top view of pink pigment development in presence of extract].

In addition to all data regarding radial growth response to the extracts (which “quantifies” inhibition or promotion of growth), it is important to note that phenotypic changes were observed at different concentrations that might further elucidate the potential of the tested extract—see Table 4 and Figure 3, Figure 4, Figure 5 and Figure 6.

**Figure 7:**
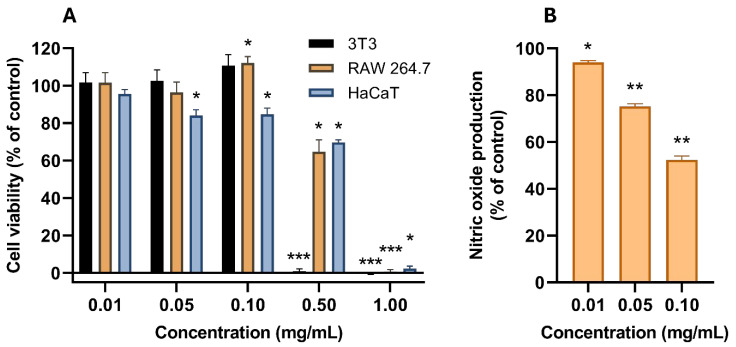
(**A**) Cell viability (in percentage of growth control) of AAG ethanol extract increasing concentrations (0.01, 0.05, 0.1, 0.5, and 1 mg/mL) in three mammalian cell lines (3T3, RAW264.7, and HaCaT). (**B**) In vitro NO production (in percentage of LPS-stimulated control in the absence of other agents) from LPS-stimulated RAW264.7 cells in the presence of non-cytotoxic concentrations of AAG ethanol extract. Asterisks indicate statistically significant differences, calculated using a one-sample *t*-test: *—*p*-value < 0.05; **—*p*-value < 0.01; ***—*p*-value < 0.001.

Cell viability was assessed in three mammalian cell lines (Figure 7). Mouse fibroblasts (3T3) demonstrated a steep dose-response shift from 100% to 0% cell viability between 0.1 and 0.5 mg/mL; at 0.1 mg/mL, a non-statistically significant trend of cell viability above 100% was noted, representing a hormesis-like induction of growth at sub-toxic concentrations. The same hormesis-like response was seen in mouse macrophages (RAW264.7), with a statistically significant (*p*-value < 0.05) increase in cell viability at 0.1 mg/mL, although in this cell line the decrease of cell viability was not so steep, with 0.5 mg/mL still presenting more than 60% cell viability. However, 1 mg/mL was also completely cytotoxic. The human keratinocytes (HaCaT) only retained complete viability at 0.01 mg/mL; 0.05–0.5 mg/mL produced a statistically significant (*p*-value < 0.05) loss of cell viability, although it remained above 50%, and 1 mg/mL produced a complete loss of viability.

Non-cytotoxic concentrations of AAG ethanol extract were tested as NO production inhibitors in LPS-stimulated RAW264.7 as a model of anti-inflammatory activity. A dose-dependent reduction in NO production was achieved with increasing doses of AAG ethanol extract, ranging from a 6% reduction (*p*-value < 0.05) at 0.01 mg/mL to a 48% reduction at 0.1 mg/mL (*p*-value < 0.01).

## 3. Discussion

In this work, a preliminary evaluation of the potential of AAG biomass for the production of an antimicrobial ingredient, which may be included in formulations targeting several industries such as (but not limited to) foods, feeds, agriculture, or healthcare, was made. The yield of extraction (1.01% W/W based on dry weight) of AAG ethanol extract is a value within that previously reported for ethanolic extractions from red seaweed [8], including *A. armata* [6,9]. This type of extraction is targeted at the secondary metabolites (“small molecules”) of the biomass, which are not the bulk of it (most of its dry weight corresponds to structural molecules, such as carbohydrates and proteins, along with inorganic molecules—salts and minerals [5,10,11,12,13]). In fact, the chemical characterization of an ethanol extract from seaweed is challenging, depending on available equipment and expertise. Ethanol is a polar organic solvent that has a very broad range of capacity of solvation in terms of small molecules, extracting from the more polar metabolites (e.g., polyphenols, low molecular weight carbohydrates, or amino acid derivatives [14,15,16]) to very low polarity molecules (e.g., sterols and other lipids [17,18]), and also pigments, including chlorophylls and carotenoids [19,20]. Unsuccessful attempts were made at quantifying carotenoids, chlorophylls, phenolic compounds, and sterols in the extracts by optimized HPLC-DAD methodologies using a series of reference compounds. Thus, an exploratory analysis by GC-MS after silylation was performed (see Figure S1 and Table 1). Noteworthy, the technique used to detect these compounds (GC-MS with derivatization by silylation) has limitations concerning the detection and characterization of molecules that are thermolabile or that have high molecular weight. In this sense, and considering the characteristics of our extract (ethanolic and prepared from dry seaweed), small macromolecules such as oligosaccharides and oligopeptides remain to be analyzed.

Among the tentatively identified compounds, there are four LMWCDs (glycerol, isosaccharino-1,4-lactone isomers 1 and 2, and floridoside) and two fatty acid derivatives (palmitic acid and oleamide). Glycerol (a 3-carbon sugar alcohol) and palmitic acid (a 16-carbon saturated fatty acid) are fundamental building blocks of triacylglycerides and other biomolecules and are regularly found in high concentration in biomass in general. Both are considerably soluble in ethanol and are therefore foreseeable components of the extract. While it is possible that they exert some sort of bioactivity in high concentrations (glycerol [21,22,23,24] and palmitic acid [25] have been described to have antimicrobial properties), it is not likely that they are responsible for the antimicrobial findings in this work precisely because of their low concentration. Oleamide is the amide derivative of oleic acid (a cis-unsaturated omega-9 18-carbon fatty acid). This compound has been previously reported in some marine organisms [26,27,28,29], but this is the first report in red seaweed that the authors are aware of. Oleamide has already been shown to possess antimicrobial activity [29,30,31,32], which may aid in explaining the results reported in this work.

One of the most significant peaks in the TIC was noticed at 15.86 min, with a proposed identity of floridoside (2-α-O-D-galactopyranosylglycerol) (Figure S2). This compound is a glycerol glycoside widely shown to be produced by red seaweed [33], which further supported the peak’s identification, and has attracted attention due to its demonstrated bioactivities (such as antioxidant, antifouling, anti-inflammatory, and immunomodulatory [33,34,35,36,37]). More importantly, floridoside has also shown some degree of antibacterial activity by potentiating the effect of antibiotics [38] and of isethionic acid in inhibiting quorum sensing [39]. Other LMWCDs can be postulated to possess biological activities such as those described for floridoside due to their chemical structure similarity. Finally, the identification of the two isomers of isosaccharino-1,4-lactone (a sugar acid derivative) prompted a careful investigation on the occurrence of this compound in nature. To the best of our knowledge, isosaccharino-1,4-lactone has only been reported as a biologically produced metabolite once, in the serum and urine of humans [40]. For that reason, replication of the detection of these two isomers in other AAG samples would confirm if they were definitely sourced from this type of biomass or a laboratory contamination. Should it be found again in AAG samples, another aspect to be investigated would be whether this compound being present in this biomass is a result of the algae’s metabolism or bioaccumulation from environmental contamination (which could be assessed using AAG sourced from a controlled environment such as aquaculture). If it was shown to be an algal metabolite, this would then be the second report of isosaccharino-1,4-lactone occurring in nature (it is widely studied under synthetic conditions). The relevance of this compound both metabolically (for AAG) and biotechnologically (in the ethanolic extract or isolated) remains to be clarified.

Finding an extract with both antimicrobial and antioxidant properties promotes its dual value as a preservative in formulations. Positive antioxidant results can still promote its use as a preservative if no antimicrobial potential is found [6]. The antioxidant activity of AAG ethanolic extract is present in Table 2. Overall, AAG ethanolic extract revealed more pronounced antioxidant activity in the assays that detect more polar antioxidant molecules. The mean LPIP value (11.5% of lipid peroxidation inhibition with 1 mg/mL of extract) was very low compared to what was expected at such a high extract concentration, since AAG extracts had already been reported to have LPIP capacity [41,42]. This strongly suggests a lack of lipophilic antioxidant molecules (contrary to what is expected for an ethanol extract), such as carotenoids, tocopherols, polyunsaturated fatty acids (PUFAs), or phytosterols [43,44]. Indeed, this is in line with the results from the GC-MS analysis, which should have detected at least some of these classes of compounds, should they exist in the ethanolic extract of AAG. Since these molecules are known to occur in red seaweeds in general [5,45,46,47,48,49,50,51], and although some have been detected in *A. armata* [5], this result suggests *A. armata* might be particularly poor in these compounds or that the extraction conditions were not favorable to their recovery.

To the best of our knowledge, there have not been any reports on FRAP activity of *A. armata* extracts prior to this work. However, Gao et al. [52] did report a FRAP value for the ultrasound-assisted extraction with ethanol from *A. taxiformis* less than half of that reported by us. In their work, however, it becomes clear that the potential of *A. taxiformis* biomass for FRAP activity is much higher than that recovered by an ethanol extraction, since other extraction methods employed by their team recovered much higher FRAP activities. Thus, it might be that *A. armata* has much higher FRAP potential in its biomass than in our ethanol extract. This may be true due to water-soluble components, such as sulfated polysaccharides (major constituents of *Asparagopsis* spp. biomass), which are not present in the ethanol extract, and are known to be able to reduce Fe^3+^ to Fe^2+^ [53,54]. Nevertheless, a mean FRAP value of 1.96 mM eq. Fe^2+^/mg extract is a relatively high value in the field of crude natural extracts from vegetal sources and renders this extract a potential for utilization as a preservative in a formula where it is employed. This is further solidified by the value of ORAC determined (15.75 µmol eq. Trolox/g extract), which is a common value for a red seaweed extract, including previous reports with *A. armata* [5,55,56]. ORAC is an antioxidant assay that heavily relies on the capacity of polar molecules, e.g., those containing labile hydrogen atoms such as those in hydroxyl groups from phenolic compounds or polyols, to transfer these protons to radical molecules; GC-MS results have shown the presence of several sugar derivatives, such as floridoside, that have been postulated and demonstrated to have antioxidant activity precisely by hydrogen donation [37,57]. Together, these results demonstrate that AAG ethanol extract possesses compounds capable of both electron-transfer (ET) and hydrogen atom transfer (HAT) mediated antioxidant activities.

In this study, multiple microbial species were used to assess AAG ethanol extract antimicrobial potential. The choice of microbial species that currently pose different threats to human, animal, and crop health, implying severe economic and human losses, allows for a comprehensive screening of AAG ethanol extract potential for multiple industries at once, assisting the creation of added value for this biomass while addressing the impending problem of antimicrobial resistance. Also, it is noteworthy that the obtainment of results ranging from growth promotion to modest inhibition to full inhibition among species that are usually grouped together (Gram-positive and Gram-negative bacteria, yeasts, and filamentous fungi) illustrates the importance of testing antimicrobial potential directly with the species of interest (often, even with specific isolates), rather than testing “model species” (typically, *S. aureus*, *E. coli,* and *C. albicans*) to infer antibacterial and antifungal activities as an umbrella concept.

The strongest inhibitory responses were observed against *Cutibacterium acnes*, *Vibrio parahaemolyticus*, *Cryptococcus neoformans*, *Malassezia furfur*, and *Fusarium graminearum*. Inhibition of *C. acnes* was particularly promising, reaching >90% at 2 mg/mL in a dose-dependent manner. Given the central role of this bacterium in acne vulgaris and the existing dermocosmetic market interest in marine-derived anti-acne agents [58], these findings support further development of AAG extracts or their fractions for skincare applications. Januário et al. [59] reported the development of an anti-*C. acnes* AAG hydroethanolic extract, which after ethyl acetate fractionation presented MIC at 1 mg/mL and MBC at 4 mg/mL. These results support the idea that polar small molecules of AAG hold potential for medical application in acne, and the ethanolic extraction may be a more cost-effective method to develop an ingredient sufficiently potent for the dermocosmetic industry. In fact, there are already some extracts from *A. armata* in the market advertised as anti-acne agents (YSaline^®^ by Algues et mer, Ushant, France, and ASPARCID P^®^ by Exsymol, Monaco, Monaco) [5]; however, these are the first scientific reports of this activity—which is an important step towards deciphering the mechanism of action and therefore further understanding both the potential of the seaweed and novel therapeutic targets of the disease.

The extract also displayed strong inhibition of *V. parahaemolyticus*, with a steep dose–response transition to full inhibition between 1 and 2 mg/mL. This species is responsible for enormous economic losses associated with animal diseases in aquaculture [60], and AAG ethanol extract has been previously shown to be able to increase shrimp survival upon bacteriological challenge with this pathogen [6]. Our results (Figure 1) confirm that a likely mechanism of action of the AAG ethanol extract in the increase of survival of infected shrimp is the reduction of bacterial load/viability, since we found 2 mg/mL of extract to be the MIC for *V. parahaemolyticus*. Bansemir et al. also found antibacterial activity of AAG against another pathogenic *Vibrio* species (*V. anguillarum*) [61], although with a different extraction solvent. Further studies pursuing deeper insight into the antibacterial mechanism of action of AAG ethanol extract against *V. parahaemolyticus* can further elucidate both the seaweed’s potential and the disease’s novel therapeutic targets.

Among yeast-like fungi, *C. neoformans* and *M. furfur* presented MIC at 2 mg/mL (Figure 2). Strengthening our results, Genovese et al. [62] reported the activity of the ethanol extract of *A. taxiformis* against *C. neoformans* and *C. gatii*, with MIC found between 1.56 and 3.12 mg/mL, depending on the strain. In the case of *M. furfur*, research on the efficacy of red seaweed extracts is scarce, and alcoholic extracts from several red seaweeds have been reported to lack activity against this fungus [63]. Because these fungi are significant human and veterinary pathogens, and antimycotic resistance is an emerging concern [64,65], this result is an important precedent to encourage further research with AAG biomass in these fields. Among filamentous fungi, *Fusarium graminearum* was the most susceptible of those tested: its growth was inhibited by 40% (Table 3), and its orange pigmentation was severely inhibited as well (Table 4 and Figure 4). Being the causative agent of Fusarium head blight (FHB), it causes massive economic losses by impacting plant growth and rendering what’s left unusable due to contamination with mycotoxins (toxic for livestock and humans alike) produced by the fungus [66,67,68,69,70,71]. Toledo et al. have demonstrated that the ethanolic extract from AAG biomass was able to inhibit spore germination in *F. oxysporum* at 1 mg/mL, showcasing the potential of AAG ethanol extract for different agricultural applications against this genus of phytopathogens. Although growth inhibition as measured by GRSDF was only moderate, the inhibition of pigmentation (Table 4/Figure 4) is an important feature of the extract; in fact, the most likely pigments that give *F. graminearum* its light and dark orange colors (which disappear upon exposure to AAG ethanol extract) are aurofusarin and rubrofusarin, and both have been associated to *F. graminearum* production of mycotoxins (aurofusarin is also considered a neglected mycotoxin) [72]. That being the case, which requires further studies for validation, our results might imply that AAG is capable of modulating the phenotype of this species, potentially towards a less toxic or virulent one.

A second group of microorganisms (*Staphylococcus aureus*, *S. epidermidis*, and *Candida albicans*) displayed “mixed” responses (Figure 1 and Figure 2), where low extract concentrations stimulated growth while higher concentrations inhibited it. Despite some authors describing a certain level of anti-*S. aureus* activity in AAG extracts [73,74,75], in the present study the ethanol extract of AAG produced a mixed-response curve (Figure 1) with increasing concentration of extract, and regardless of how much it was increased, the antibacterial effect reached only moderate levels (35.4% at 2 mg/mL). At lower concentrations (0.01 and 1 mg/mL), it even promoted bacterial growth. The mixed-response curve is likely a consequence of the crude nature of the extract, with multiple compounds’ concentrations increasing with total extract increase, and therefore the balance between the dose-response of the bacterium to growth promoters and growth inhibitors oscillating. While MIC values were reached at 2 mg/mL for *S. epidermidis* and *C. albicans*, the stimulatory effect observed at lower concentrations highlights the need for fractionation and identification of the active compounds before any safe application can be considered. In the case of C. albicans, the presence of antimicrobial compounds in AAG is further supported by Salvador et al. [73].

In contrast, inhibition of *Bacillus cereus*, *Escherichia coli*, *Aspergillus fumigatus*, and *Stemphylium vesicarium* was weak or inconsistent. For *B. cereus* and *E. coli*, inhibition was mild and substantially lower than reported for extracts obtained with other solvents [73,74], suggesting ethanol may be suboptimal for extracting the relevant antibacterial compounds. There is, however, one utility to this discovery: it might be that AAG ethanol extract is safe for co-administration with supplements of *B. cereus* where it is being used as a beneficial probiotic, biocontrol agent, or biofertilizer [76,77,78,79,80,81,82,83,84]. *A. fumigatus* showed only slight growth inhibition (19.5% GRSDF, Table 3), although a more interesting effect was the reduction of spore formation (Table 4/Figure 3), which may justify further investigation, especially in a setting of increasing antifungal treatment resistance [85]. This ability to reduce or inhibit spore formation has seldom been reported; it was, nonetheless, found with *A. fumigatus* in a study by Rogawansamy et al. [86] testing two common multipurpose industrial disinfectants (Cavicide^®^ and Virkon^®^), 70% ethanol, vinegar (4.0%−4.2% acetic acid), and a plant-derived compound (tea tree (*Melaleuca alternifolia*) oil), as well as with *A. niger* exposed to natural extracts (ethanolic as well) from the lichen *Pseudevernia furfuracea* [87]. Anti-*Aspergillus* activity has previously been reported for an extract containing oleamide [30], which might indicate that the presence of this compound in AAG ethanol extract can be responsible for such slowdown. *S. vesicarium* also exhibited low growth inhibition (Table 3), with partial pigment changes of unclear biological significance. Taken together, these results suggest that crude ethanolic extracts of AAG are unlikely to be effective against these organisms without further optimization.

Since the growth of *S. uberis* was seemingly promoted by AAG ethanol extract (Figure 1), it is apparent that this extract is inappropriate for use as an agent to prevent or treat bovine mastitis, nor should it be employed in feed or veterinary product formulations intended for use in dairy cattle. Finally, in the case of *Tricophyton rubrum*, growth (Table 3) was virtually unaltered (GRSDF of −1.574 µm/h [−2.317; −0.8308]). However, its phenotype did change (Table 4/Figure 6), with the extract-treated fungus lacking its bottom orange pigmentation and a pinkish one surging on top. There are three pigments associated with *T. rubrum* (xanthomegnin, viopurpurin, and vioxanthin), and the three have been considered virulence factors by different mechanisms [88]. Also, color change in colonies of *T. rubrum* has been reported to be common in the presence of pH changes, with yellowish color being present in acid conditions and reddish color in alkali ones [88]. Whether the change in pigmentation induced by our extract is due to changes in pH caused by the extract itself and/or altered fungal metabolism, or whether it is a sign of different pigment production, is unknown and should be the object of future research. In any case, given the role in pathology that *T. rubrum*’s pigments play, one might wonder if there is an altered virulence upon exposure to AAG ethanol extract, and that is certainly something that is worth being clarified.

Cell viability of three mammalian cell lines (3T3 mouse fibroblasts, RAW264.7 mouse macrophages, and HaCaT human keratinocytes) was studied when subjected to AAG ethanol extract (Figure 7). Murine cell lines (3T3 and RAW264.7) produced a hormetic dose response, with cell viability being higher than control at 0.1 mg/mL before dropping to complete cell death at 1 mg/mL. In HaCaT, this response was not detected, and instead, a less defined S-shape was found, with a more gradual decrease of viability across tested concentrations. This human cell line was also slightly more susceptible to the extract, since lower than control cell viability was detected from 0.05 mg/mL. Overall, however, the maintenance of cell viability above 80% in the three cell lines with up to 0.1 mg/mL provides a positive preliminary indicator of sufficient safety to find concentrations that are both harmless to human and animal tissues while maintaining desirable bioactivities. For instance, at 0.1 mg/mL, significant NO production inhibition (up to 48%) was found in the LPS-stimulation of RAW264.7 macrophages assay (Figure 7). This activity may be due to floridoside, which has had anti-inflammatory activity reported before [35]. Also, the amount of LMWCDs potentially present in the extract may encompass molecules such as steroidal and phenolic glycosides, known to potentially possess anti-inflammatory activity [89]. Studies on the anti-inflammatory activity of red seaweed are generally related to water-soluble metabolites, namely polysaccharides and amino acids, or hydrophobic metabolites such as fatty acids and steroids [90]; polar metabolites such as phenolic compounds and glycosides are not frequently reported in red algae anti-inflammatory activity studies.

Our work contributes significantly to the dataset of bioactivities of *A. armata* with data that is innovative in terms of the molecules identified in the ethanolic fraction of this seaweed and in the set of pathogens assayed. Future research should address both the chemical constituents responsible for the activities herein reported and the molecular mechanisms underlying these same activities. Also, in vivo safety data must be made available to validate whether this ingredient can be used for animal (including human) applications.

## 4. Materials and Methods

### 4.1. Seaweed Biomass Harvesting and Processing

*Asparagopsis armata*’s gametophyte, hereafter referred to as AAG, was harvested from Reserva Natural das Berlengas in Portugal (41.054826, −8.656865) on 28 June 2019. The collected biomass was cleaned and sorted for epibionts and frozen at −20 °C until further usage. Then, the biomass was dried in a wind tunnel at 35 °C, ground to a flour-like powder with a particle size of 150 ± 50 µm, and vacuum stored at room temperature and in the dark until use.

### 4.2. Ethanol Solid-Liquid Extraction

A solid-liquid extraction (SLE) with ethanol as a solvent was performed using dry AAG biomass. In this method, 100 g of the algal biomass was mixed with 1 L of absolute ethanol and stirred for 20 min, following a previously optimized SLE protocol [6]. The resultant mixture was filtered using Whatmann™ n°1 paper filters (Cytiva, Marlborough, MA, USA) to remove any suspended solids, and the ethanol extract was collected. The extract was subsequently evaporated using a rotary evaporator (with a water bath temperature of 35 °C) under reduced pressure until dryness in a previously weighed round-bottom flask so that yield could be calculated. The extraction was performed in triplicate.

### 4.3. Extract Chemical Characterization (Gas Chromatography-Mass Spectrometry, GC-MS)

An exploratory analysis was conducted to tentatively identify secondary metabolites in the dry ethanol extract of AAG using GC-MS and following trimethylsilyl (TMS) derivatization. The protocol used was adapted from Araújo et al. [91]. Briefly, 20 mg of dry extract were dissolved in 250 µL of pyridine, and then 250 µL of N,O-bis(trimethylsilyl)trifluoroacetamide (BSTFA) and 50 µL of chlorotrimethylsilane (TMCS) were added. The mixture was incubated at 70 °C for 30 min. The chromatographic analysis was conducted using Helium C-60 (Gasin, Coimbra, Portugal) as a carrier, at 1.0 mL/min, in a capillary column Rxi-5Sil MS (30 m × 0.25 mm × 0.25 µm) from RESTEK, on an EVOQ 436 GC system (Bruker Daltonics, Fremont, CA, USA) coupled to a SCION Triple Quadrupole mass detector. The sample was diluted (1:50), and 2 µL were injected in split mode (1:20 ratio) using an injector set to 250 °C (held for 20 min). The oven temperature was set at 70 °C for 2 min, increased to 250 °C at a rate of 15 °C/min, kept for 2 min, then increased to 300 °C at a rate of 10 °C/min, and then held for 5 min. The entire separation run took 26 min. The MS detector was operated in the EI mode (70 eV) with an EI temperature of 270 °C, a transfer line temperature of 280 °C, and a manifold temperature of 40 °C. A mass-to-charge ratio range of 50 to 500 m/z was used for data collection in full scan mode.

### 4.4. Extract’s Antioxidant Activity

#### 4.4.1. Ferric Reducing Antioxidant Potential (FRAP)

The FRAP of the ethanolic extract was determined using a modified protocol based on Ferreira et al. [3]. To prepare the working reagent (WR), 10 volumes of 300 mM acetate buffer (pH 3.6), 1 part of 10 mM TPTZ (2,4,6-tri(2-pyridyl)-s-triazine (Sigma Aldrich, St. Louis, MO, USA) in 40 mM HCl, and 1 part of 20 mM FeCl2 (ferric chloride) were combined daily. The assay was carried out in a 96-well microplate with eight wells per condition, where 8 μL of each standard/sample’s concentration per well, or 8 μL of DMSO as a vehicle control, was pipetted. In four of the eight wells, 272 μL of WR was added, and in the other four wells, 272 μL of water was added as blanks. FeSO4 (1 mM) was used as a standard/positive control. After incubating for 60 min at room temperature in the dark, the absorbance was measured at 593 nm (EPOCH 2 microplate reader, BioTek^®^ Instruments, Winooski, VT, USA). The antioxidant activity was calculated by subtracting the blanks from the samples, as well as the vehicle control, and then dividing the resulting value by that of the positive control (Fe^2+^ standard) minus the negative control. The assay was performed in triplicate, and the results were expressed in mM eq. Fe (II)/mg extract.

#### 4.4.2. Oxygen Radical Absorption Capacity (ORAC)

The ORAC of the ethanol extract obtained from AAG was assessed following the methods of Félix et al. [92] and Dávalos et al. [93]. The antioxidant activity was compared with that of Trolox (6-hydroxy-2,5,7,8-tetramethylchroman-2-carboxylic acid). To prepare a standard curve, a Trolox stock solution was made in phosphate buffer (75 mM, pH 7.4) and diluted to concentrations ranging from 0.5 to 8 µM. The extract was tested at 1 mg/mL (diluted in 75 mM phosphate buffer). As an oxidant, AAPH (2,2′-Azobis(2-methylpropionamidine) dihydrochloride) reagent (Sigma, Darmstadt, Germany) at 12 mM was prepared, and a fluorescein solution at 70 nM was used as a “sensor”. Then, 20 μL of each sample and 120 μL of the fluorescein solution were added in a 96-well black microplate (Greiner, Kremsmünster, Austria) with 4 wells/condition. Phosphate buffer at 75 mM was used as a control. Initially, the fluorescence (excitation wavelength of 485 nm and emission wavelength of 525 nm) was read for 15 min with a 1 min interval at 37 °C using a microplate reader (Synergy H1, Biotek, Winooski, VT, USA). After that, 60 μL of AAPH at 37 °C was added, and the fluorescence was read for 80 min with 1 min intervals. The area under the curve (AUC) was calculated, and the sample’s antioxidant activity was compared with that of Trolox. Results were expressed as μmol of Trolox equivalents per gram of extract (μmol TE/g ext), and the assay was performed in triplicate.

#### 4.4.3. Lipid Peroxidation Inhibitory Potential (LPIP)

The Lipid Peroxidation Inhibitory Potential (LPIP) of the ethanol extract was evaluated by a method adapted from Reboleira et al. [94]. First, a linoleic acid suspension was prepared by vortexing a sample of linoleic acid (final concentration: 20 mM) in the adequate volume of suspension buffer (Tween 20 at 5.6 mg/mL in phosphate-buffered saline (PBS) at 20 mM, pH 7.1). After, the first reaction (auto-oxidation of the linoleic acid) was prepared by combining 25 µL of a given sample (the extract at different concentrations—10, 1, and 0.1 mg/mL; the standard curve’s solutions—α-tocoferol at 10, 1, 0.1, 0.01, and 0.001 mg/mL—or vehicle solutions) with 125 µL of the linoleic acid suspension (in triplicate) and 100 µL of PBS (same as above) in 2 mL microtubes. As blanks, tubes with extract but with suspension buffer instead of linoleic acid suspension were used (to determine peroxides native to the extract and subtract them from the final result). The positive control (maximum peroxidation) was prepared using 25 μL of extract vehicles (DMSO in PBS at different concentrations) and 125 μL of linoleic acid suspension (along with 100 μL of PBS), and as a negative control, 25 μL of vehicle and 125 μL of suspension buffer with 100 μL of PBS. The tubes were capped and incubated at 37 °C for 48 h in the dark. The experiment was performed in triplicate.

The peroxides formed were then quantified using the thiocyanate method. First, a working reagent was prepared by mixing 47 parts of ethanol 75% (*v*/*v*) in water with 1 part of ammonium thiocyanate at 30% (*w*/*v*). Then, 20 μL from each auto-oxidation tube were added (in triplicate) to a tube (×3) with 960 μL of working reagent. Finally, 20 μL of iron (II) chloride at 20 mM prepared in HCl at 3.5% (*w*/*v*) was added to each tube. After homogenization, the absorbance at 500 nm was read for each tube. LPIP was expressed as a percentage of inhibition of peroxidation.

### 4.5. Extract’s Antimicrobial Activity

#### 4.5.1. Antibacterial and Anti-Yeast Activity

The ethanol extract of AAG was assessed for its antibacterial and anti-yeast activity using the microdilution technique [92,95,96,97]. The following microorganisms were tested: *Bacillus cereus* DSM 31; *Staphylococcus aureus* DSM 1104; *Staphylococcus epidermidis* DSM 20044; *Escherichia coli* DSM 1103; *Cutibacterium acnes* DSM 1897; *Vibrio parahaemolyticus* (isolated from infected *Litopenaeus vanammei*); *Streptococcus uberis* DSM 20569; *Candida albicans* DSM 819; *Cryptococcus neoformans* DSM 6973; and *Malassezia furfur* CBS 7854. The microorganisms were grown in a specific culture medium and for a specific time period, as per the Clinical and Laboratory Standards Institute (CLSI) recommendations (Table 5). Each species was subjected to a microdilution assay in a specific culture medium and for a defined period of incubation (Table 5).

On the day of the experiment, a new inoculum stock solution was created in saline solution (0.85% *w*/*v* NaCl) by dissolving colonies from solid media growth until a density of 0.5 McFarland was achieved, which was measured using a portable turbidimeter (Grant-bio DEN-1B, Grant Instruments, Royston, UK). This solution was diluted accordingly to achieve a working solution of inoculum so that after microplate assembly the final concentration of Colony Forming Units (CFU) in the microplate wells was 5 × 10^5^ CFU/mL for aerobic and microaerophilic bacteria, 1 × 10^6^ for anaerobic bacteria, and 1.5 × 10^3^ CFU/mL for yeast. Then, the extract’s multiple concentrations to be tested (2, 1, 0.1, and 0.01 mg/mL) were prepared by dissolving a stock solution of extract (100 mg/mL in DMSO) in the assay’s culture medium, following which the solutions were filtered through a 0.22 µm cellulose acetate syringe filter (VWR International, LLC, Carnaxide, Portugal) to ensure the extract’s possible microbial contaminants did not interfere with the assay. The experiment was conducted using sterile round-bottom microplates (Thermo Scientific, Waltham, MA, USA).

The microplates were assembled by pipetting 190 µL of medium (simple—in the case of controls—or containing extract) and 10 µL of inoculum working solution (or saline in the case of extract blanks and the assay’s control of sterility). Four wells per condition were used. An additional group of four wells containing 2 µL of antimicrobial agents were used as inhibition control (refer to Table 5). “Full growth” controls were also included using either simple medium or medium containing the extract’s vehicle (DMSO) at appropriate concentrations. After incubation, the optical density was measured at 625 nm in a microplate reader (Epoch2, BioTek, Winooski, VT, USA), and the percentage of growth inhibition was calculated. The assay was performed in triplicate.

#### 4.5.2. Antifungal Activity (Filamentous Fungi)

The activity of extracts against filamentous fungi may be tested by several different assays [98]. One of the most straightforward is the Poisoned Food Technique (PFT) originally developed by Grover and Moore [99], which was used to evaluate the ethanolic extract of AAG after some modifications. In this method, plugs (5 mm ø) of fungi actively growing in solid media are inoculated into another solid media containing the extract. The growth and phenotype of the fungi under these conditions are compared to control conditions to assess the effect of the extract on the fungal species. The antifungal activity of the ethanol extract was evaluated against *Aspergillus fumigatus* DSM 819, *Fusarium graminearum* CBS 389.62, *Stemphylium vesicarium* CBS 124751, and *Tricophyton rubrum* CBS 113208. Initially, each species was cultured in Potato Dextrose Agar (PDA) under optimal temperature and light conditions.

Sufficient PDA was prepared to set up several 6-well microplates, with each well containing 2.5 mL of media. Various aliquots of PDA were treated differently, including some that were poured directly into the wells, others that were mixed with DMSO in appropriate amounts to serve as controls for the vehicle concentrations present in the extracts, and still others that were used to prepare different concentrations (2, 1.5, 1, 0.1, and 0.01 mg/mL) of PDA-containing extract. The extract, dissolved in 10% DMSO in saline solution, or DMSO alone was filtered through a 0.22 µm cellulose acetate syringe filter and added to the autoclaved liquid PDA just before solidification to avoid thermal degradation. For each concentration of each replicate of extract, three wells were plated. Once the 6-well plates had cooled to room temperature, 5 mm plugs of fungal inoculum were placed in the center of each well. The plates were then sealed with Parafilm M^®^ on the sides to prevent medium dehydration during the assay. Fungal growth was monitored twice daily until the radial growth had reached the borders of at least one condition for each species. During such period, radial growth in millimeters was measured, and phenotypic annotations were made along with photographs.

### 4.6. Cell-Based Assays

Mouse fibroblasts (3T3 DSMZ–ACC 173) and macrophages (RAW264.7, ATCC-TIB 71) and human keratinocytes (HaCaT, CLS catalog number 300493) were grown and maintained according to the manufacturer’s instructions. Briefly, 96-well microplates inoculated with 5 × 10^4^ cells/well (3T3 and RAW264.7) or 8 × 10^4^ cells/well (HaCaT) were grown in Dulbecco’s modified Eagle medium (DMEM) (Sigma, Germany) supplemented with 10% FBS (Biowest, Nuaillé, France) at 37 °C in 5% CO_2_ for 24 h. Then, the culture media was replaced by 100 µL of DMEM, 10% FBS, plus 100 µL of extract solution in phosphate-buffered saline (PBS). A dose-response curve (final concentration of AAG extract in wells: 0.01, 0.05, 0.1, 0.5, and 1 mg/mL) was performed. After 24 h, the medium containing extract was removed, and 100 µL of PBS were used to wash the cells.

The 3T3 cell viability was evaluated using the Neutral Red method [92,100]. After washing, the microplates were incubated with 100 µL of DMEM, 5% FBS, without phenol red and supplemented with Neutral Red solution (40 µg/mL in PBS) (Sigma, Germany) at 37 °C in 5% CO_2_ for 4 h. After washing with PBS, 100 µL of desorption solution (glacial acetic acid, ultrapure water, and absolute ethanol 1:49:50 *v*/*v*/*v*) was used to achieve complete homogenization. The absorbance was read at 540 nm in a microplate spectrophotometer. In the case of RAW264.7 and HaCaT, cell viability was determined using the 3-(4,5-dimethylthiazol-2-yl)-2,5-diphenyl tetrazolium bromide (MTT) method, adapted from Bahiense and colleagues [101]. After washing, the microplates were incubated with 100 µL of DMEM with 5% FBS, without phenol red and supplemented with MTT solution (0.5 mg/mL in PBS) (Sigma, Germany). Incubation was carried out at 37 °C in 5% CO_2_ for 4 h and then PBS was used to wash. For complete solubilization of formazan, 100 µL of DMSO was added, and the plates were agitated in the absence of light. Finally, the absorbance was read at 570 nm. Data presented is the result of 3 independent replicates and is expressed as a percentage of the control (which was prepared with the corresponding percentage of vehicle—DMSO).

Nitric oxide (NO) production in mouse macrophages (RAW264.7 cells) was assayed as a proxy for anti-inflammatory potential using the Griess diazotization reaction, according to Félix et al. [92]. Only the non-cytotoxic concentrations of AAG ethanol extract (0.01, 0.05, and 0.1 mg/mL) were tested. The microplates were seeded with 1 × 10^5^ cells/well and incubated at 37 °C in 5% CO_2_ for 24 h. The medium was removed, and cells were then exposed to the extracts (100 µL prepared in DMEM with 5% FBS without phenol red) for 6 h, at which time 100 µL of a 3 µg/mL LPS (lipopolysaccharide) from *Escherichia coli* (Sigma, Germany) solution was added for an additional 22 h. Finally, 150 µL of the supernatants of the cells was mixed with 50 µL of Griess reagent (Sigma, Germany). The absorbance was measured at 540 nm after 15 min at room temperature using a microplate spectrophotometer, and 3 independent assays were performed.

All data concerning cell-based assays is presented in graphics along with the significance of the one-sample *t*-test (GraphPad Prism 8.4.3, Boston, MA, USA) conducted with a test value of 100.

## Figures and Tables

**Table 1:** Tentative identification of compounds in the silylated ethanolic extract of AAG by GC-MS. Relative abundance is calculated from the sum of peak heights for all identified peaks. Identification levels are indicated: L1—identification of the metabolite of interest compared to a chemical reference standard under identical analytical conditions within the same laboratory; L2—putatively annotated compounds through MS similarity with the NIST database; L3—putatively characterized compound classes: spectral and/or physicochemical properties consistent with a particular class of organic compounds [7]. Rt—Retention time; Exp.—Experimental; Lit.—Literature; TMS—Trimethylsilyl groups; LMWCD—Low Molecular Weight Carbohydrate Derivative; n.a.—not applicable; n.av.—not available.

Rt	Relative Abundance (%)	Exp. Kovats	Compound Names	Lit. Kovats	Identification Level
8.37	34.33		Glycerol (3TMS)		L1
11.60	0.70	1695	LMWCD	n.a.	L3
11.66	0.52	1702	LMWCD	n.a.	L3
11.76	0.47	1714	LMWCD	n.a.	L3
11.83	0.38	1721	LMWCD	n.a.	L3
11.96	1.93	1736	Isosaccharino-1,4-lactone (3TMS) isomer 1	n.av.	L2
12.05	6.84	1747	Isosaccharino-1,4-lactone (3TMS) isomer 2	n.av.	L2
13.38	0.57	1901	LMWCD	n.a.	L3
13.62	0.57	1930	LMWCD	n.a.	L3
13.90	0.63	1961	LMWCD	n.a.	L3
14.48	0.83		Palmitic acid (TMS)		L1
15.53	0.91	2150	LMWCD	n.a.	L3
15.86	42.93	2189	Floridoside (6TMS)	2180	L2
16.17	0.98	2225	LMWCD	n.a.	L3
16.28	1.06	2238	LMWCD	n.a.	L3
16.57	1.13	2272	LMWCD	n.a.	L3
17.32	5.22		Oleamide		L1

**Table 2:** Antioxidant activity of AAG ethanolic extract measured by FRAP, ORAC, and LPIP methodologies.

Assay	Antioxidant Activity (Mean ± SEM)
FRAP (mM eq. Fe^2+^/mg extract)	1.96 ± 0.17
ORAC (µmol eq. Trolox/g extract)	15.75 ± 1.34
LPIP (% inhibition at 1 mg extract/mL)	11.5 ± 8.9

**Table 3:** Control growth rate (maximum growth in the absence of extract) in µm/h, Growth Rate Slowdown Factors (GRSDF) in µm/h and percentage of growth inhibition for the four species of filamentous fungi tested. Results are expressed as the mean followed by the 95% Confidence Interval in square brackets. g.s.—growth stimulation.

Species	GRSDF (Mean [95CI], in µm·h^−1^, and % Inhibition)	Control Growth Rate (Mean [95CI], in µm·h^−1^)
*A. fumigatus*	27.71 [23.56; 31.86] (19.5%)	142.2 [138.8; 145.7]
*F. graminearum*	66.92 [55.94; 77.9] (40%)	167.3 [163.8; 170.7]
*S. vesicarium*	19.77 [16.12; 23.42] (19.6%)	100.9 [98.51; 103.2]
*T. rubrum*	−1.57 [−2.32; −0.83] (g.s.)	34.91 [34.37; 35.45]

**Table 4:** Summarizing table of the visual aspect of the four species of filamentous fungi tested under normal conditions (“Normal phenotype”) and exposed to the ethanol extract from AAG (“Phenotypic alterations”). In square brackets, the link between the description and a photograph of the fungal growth can be found.

Species	Normal Phenotype	Phenotypic Alterations
*A. fumigatus*	White mycelium, with a centered dark pigmentation ring (spores) [Figure 3a]	Inhibition of sporulation at 1–2 mg/mL [Figure 3b]
*F. graminearum*	Dark orange mycelium, with yellow borders; yellow aerial hyphae at center (plug) [see Figure 4a]	Marked reduction of pigmentation, attributable to extract (1–2 mg/mL); also, weakened mycelium and absent aerial hyphae, but attributable to vehicle (DMSO). [see Figure 4b–d]
*S. vesicarium*	Top view: white mycelium and aerial hyphae; Bottom view: dark pigmentation concentric ring in mycelium [see Figure 5a,b]	Partial inhibition of pigmentation at 1.5–2 mg/mL [see Figure 5c]
*T. rubrum*	Top view: white mycelium and aerial hyphae; Bottom view: orange pigmentation concentric ring in mycelium [see Figure 6a,b]	1–2 mg/mL: Inhibition of bottom orange pigmentation and stimulation of top pinkish pigmentation [see Figure 6c]

**Table 5:** Growth conditions prior to and during microdilution assay for the 10 species tested, as recommended by CLSI. CM—Culture Medium; gT&T—Growth Time and Temperature; AA—Antimicrobial agent (used as standard) and respective final concentration in the well; TSA—Tryptic Soy Agar; MHB II—Mueller Hinton Broth II Cation-Adjusted; CIP—Ciprofloxacin; TSYEA—Tryptone Soy Yeast Extract Agar; TET—Tetracycline; CBA—Columbia Blood Agar; BHI—Brain Heart Infusion; VAN—Vancomycin; CTX—Cefotaxime; PDA—Potato Dextrose Agar; RPMI—Roswell Park Memorial Institute 1640 Medium; AMB—Amphotericin B; SDB—Sabouraud Dextrose Broth.

Species	Before Assay		During Assay		
	CM	gT&T	CM	gT&T	AA
*Bacillus cereus*	TSA	24 h; 35 °C	MHB II	20 h; 35 °C	CIP 4 µg/mL
*Staphylococcus aureus*	TSYEA	24 h; 35 °C	MHB II	20 h; 35 °C	TET 16 µg/mL
*Staphylococcus epidermidis*	TSYEA	24 h; 35 °C	MHB II	20 h; 35 °C	TET 16 µg/mL
*Escherichia coli*	TSA	24 h; 35 °C	MHB II	20 h; 35 °C	CIP 4 µg/mL
*Cutibacterium acnes*	CBA	72 h ^a^; 35 °C	BHI ^b^	48 h ^a^; 35 °C	VAN 4 µg/mL
*Vibrio parahaemolyticus*	TSA ^c^	24 h; 35 °C	MHB II ^c^	20 h; 35 °C	CTX 4 µg/mL
*Streptococcus uberis*	CBA	24 h ^d^; 35 °C	BHI ^b^	20 h ^d^; 35 °C	CIP 4 µg/mL
*Candida albicans*	PDA	24 h; 35 °C	RPMI	48 h; 35 °C	AMB 4 µg/mL
*Cryptococcus neoformans*	PDA	48 h; 25 °C	RPMI	72 h; 35 °C	AMB 4 µg/mL
*Malassezia furfur*	PDA ^e^	72 h; 30 °C	SDB ^f^	72 h; 35 °C	AMB 4 µg/mL

^a^—anaerobic (grown with Anaerocult^®^ A). ^b^—containing 1% (*w*/*v*) glucose. ^c^—containing 3% (*w*/*v*) NaCl. ^d^—microaerophilic (grown with Anaerocult^®^ C). ^e^—containing 1% (*v*/*v*) olive oil emulsified prior to solidification. ^f^—containing 1% (*v*/*v*) Tween 80.

## Data Availability

The data underlying this article will be shared on reasonable request to the corresponding author.

## Supplementary Materials

The following supporting information can be downloaded at: https://www.mdpi.com/article/10.3390/ijms262311358/s1.
