# Supplementary material for: On the Antimicrobial Potential of Asparagopsis armata’s Ethanol Extract: A New Multiple-Industry Bio-Product?
Source: Int J Mol Sci. 2025 Nov 24;26(23):11358. doi: 10.3390/ijms262311358 (PMC12692125; doi:10.3390/ijms262311358)
Supplement: Supplementary file 1 [file ijms-26-11358-s001.zip › ijms-3954434-supplementary.pdf]

Supplementary Material

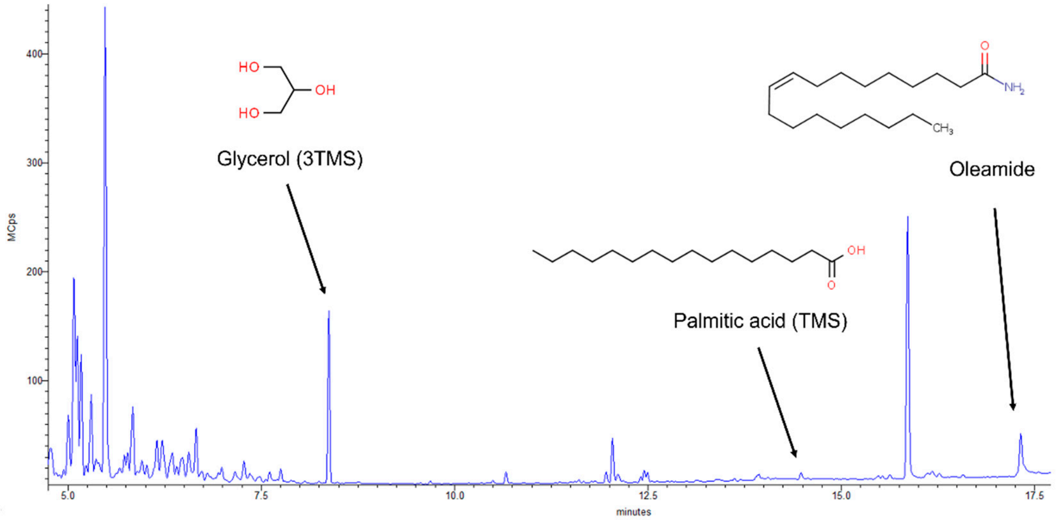

Figure S1 – Total Ion Chromatogram (TIC) obtained for the silylated ethanolic extract of AAG by GC-MS. Data acquisition was performed in the mass-to-charge ratio range of 50-500 m/z. The three compounds illustrated are those identified by comparison to a chemical reference standard under identical analytical conditions within the same laboratory.

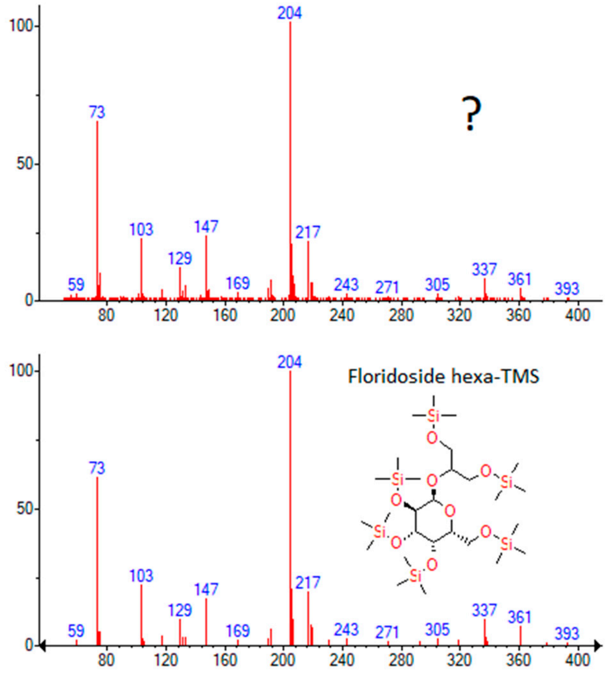

Figure S2 – Mass spectra of the peak eluted at 15.86 min (top) and of floridoside hexa-TMS (bottom).

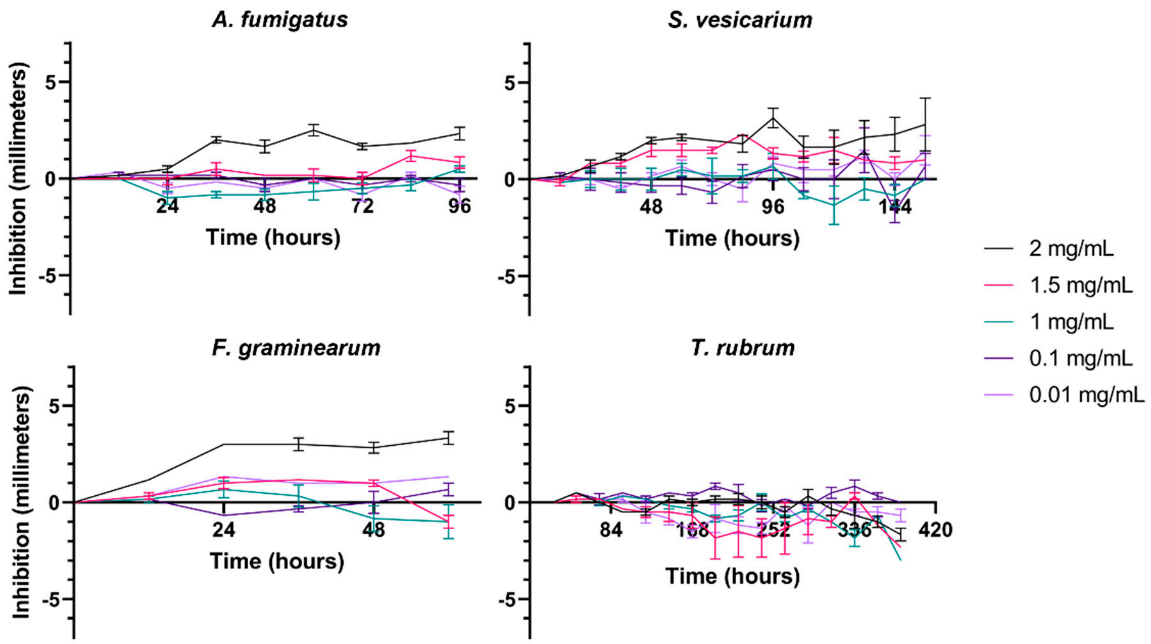

Figure S3 – Size of radial inhibition (in millimeters) with growth time for *Aspergillus fumigatus, Fusarium graminearum, Stemphylium vesicarium* and *Tricophyton rubrum* in the presence of AAG ethanol extract at five different concentrations (2, 1.5, 1, 0.1 and 0.01 mg/mL). Error bars in each datapoint are the calculated SEM (n=3).
